# Morpho-Physio-Biochemical and Molecular Responses of Maize Hybrids to Salinity and Waterlogging during Stress and Recovery Phase

**DOI:** 10.3390/plants10071345

**Published:** 2021-07-01

**Authors:** Umer Mahmood, Saddam Hussain, Sadam Hussain, Basharat Ali, Umair Ashraf, Shahid Zamir, Sami Asir Al-Robai, Fatima Omari Alzahrani, Christophe Hano, Mohamed A. El-Esawi

**Affiliations:** 1 Department of Agronomy, University of Agriculture, Faisalabad 38040, Pakistan; umermahmooduaf@gmail.com (U.M.); ch.sadam423@gmail.com (S.H.); bali@uaf.edu.pk (B.A.); zamir757@gmail.com (S.Z.); 2 College of Agronomy and Biotechnology, Southwest University, Chongqing 400715, China; 3 College of Agronomy, Northwest A&F University, Yangling 712100, China; 4 Department of Botany, Division of Science and Technology, University of Education, Lahore 54770, Pakistan; umairashraf2056@gmail.com; 5 Department of Biology, Faculty of Science, Albaha University, Al Baha 65527, Saudi Arabia; salrobai@bu.edu.sa (S.A.A.-R.); drfatimaomari@gmail.com (F.O.A.); 6 Laboratoire de Biologie des Ligneux et des Grandes Cultures (LBLGC), INRAE USC1328, Université d’Orléans, 28000 Chartres, France; hano@univ-orleans.fr; 7 Botany Department, Faculty of Science, Tanta University, Tanta 31527, Egypt

**Keywords:** plant growth, waterlogging, antioxidant machinery, ROS production, chlorophyll pigments, salt stress

## Abstract

Maize is one of the most economically important cereal crops worldwide. Salinity coupled with waterlogging is a major challenge for successful crop production. Understanding the underlying mechanisms and impacts of individual and combined salinity and waterlogging stress on the morpho-physio-biochemical and molecular responses and oxidative metabolism of maize during stress and recovery periods is essential. The present study was carried out to assess the response of four hybrid maize cultivars viz. DK-6142, FH-1231, FH-949, and MALKA-2016 under individual and combined salinity and waterlogging conditions. The treatments comprised the control (no stress), NaCl (salinity with 10 dSm^−1^), WL (waterlogged conditions with 3 cm flooding), and NaCl + WL (combined salinity and waterlogging stress). The data regarding morpho-physiological attributes were collected at 22 days after sowing (DAS; stress phase) and 30 DAS (recovery phase). The results revealed that both stresses, either individually or in combination, substantially reduced the root-shoot length, root-shoot fresh and dry weights, leaf width, and the number of leaves per plant as well as the leaf chlorophyll (Chl) and carotenoids contents; however, the inhibitory effects were more severe in combined stresses than for individual stress factors in many cultivars. Both individual and combined stress conditions enhanced hydrogen peroxide (H_2_O_2_) accumulation, whereas the antioxidant enzyme activities, i.e., superoxide dismutase (SOD), peroxidase (POD) catalase (CAT), and ascorbate peroxidase (APX), remained higher under stress conditions compared to the control. The expression levels of antioxidant genes (*CAT* and *POD*) were also upregulated under stress conditions. All of the cultivars recovered better from individual stresses than combined stress conditions; however, the hybrid DK-6142 performed better than the other maize hybrids under stress conditions and showed faster recovery.

## 1. Introduction

Salinity coupled with waterlogging is a major challenge for successful crop production. Both of these stresses are major obstacles to the long-term sustainability of irrigated lands, efficient crop production, and farmer subsistence, especially in the Indus Basin area of Pakistan [1]. Little research has previously been carried out on plant responses and tolerance to combined salinity and waterlogging stresses. Identifying crop genotypes resistant to these stresses is thus critical for the long-term viability of agriculture in stress-prone areas.

Maize is an important cereal crop and consumed as a staple food worldwide [2], whereas salinity and waterlogging are the major constraints to its growth and productivity [3,4]. Salinity causes Na^+^ toxicity in plants and thus leads to a distinct reduction in crop growth mainly during the early growth stages [5]. The effects of salinity at the germination and seedling stages were more drastic than the lateral growth stages of field crops [6]. Salinity has been reported to impair root and shoot lengths, and the biomass of different field crops [6,7,8]. For instance, under high salt concentrations, large amounts of Na^+^ and Cl^-^ were transported to the vegetative parts of maize, which antagonistically decreased the K^+^ and Ca^2+^ levels in these plants [9]. Besides damaging the embryo and changing the signal transduction pathway, salinity lowers osmotic potential and reduces water uptake during seed imbibition [2,10]. Salinity also affects leaf initiation, internodal growth, and leaf expansion, thereby reducing shoot growth in plants [11]. Furthermore, under salinity stress, the accumulation of Na^+^ disrupts the uptake of K^+^, which reduces stomatal conductance and thus creates water-deficit conditions for plants [12]. Reduced stomatal conductance under salt stress may also lead to a reduction in photosynthetic activities [13]. Salinity also results in excessive production of reactive oxygen species (ROS) [14] and a decrease in chlorophyll contents [15].

Waterlogging also leads to substantial decreases in root respiration [16], seedling growth [17], root and shoot lengths, as well as biomass accumulation [18,19,20]. It further causes an imbalance in the plant–nutrient relationship [21]. Increased concentrations of abscisic acid under waterlogged conditions may result in stomatal closure and a decrease in the photosynthetic rate [22]. Waterlogging also induces the overproduction of ROS including hydrogen peroxide (H_2_O_2_), hydroxyl radical (OH^.^), singlet oxygen (^1^O_2_), and superoxide radical (O^−^_2_.) [23,24] and disturbs normal plant metabolism [25]. Plants have adapted complex antioxidant defensive mechanisms to protect cells from the detrimental effects of ROS [26], such as catalase (CAT), superoxide dismutase (SOD), glutathione reductase (GR), ascorbate peroxidase (APX), and peroxidase (POD). These are the major scavenging enzymes that protect plants from the deleterious effects of ROS [26,27]. Although, previous studies have reported the negative effects of salinity [6,28] and waterlogging [17,29] on various field crops, nevertheless, the combined effects of both of these stresses on the morpho-physiological and biochemical attributes of maize are not well understood. It was hypothesized that the combined occurrence of salinity and waterlogging stress might have more detrimental impacts on the maize crop than individual salinity or waterlogging stress and that the responses of different maize hybrids to these stress factors might be variable. The specific objectives of the present study are (a) to evaluate the individual as well as interactive effects of waterlogging and salinity on morpho-physiological, biochemical, and molecular responses of maize hybrids; (b) to compare the salt and waterlogging tolerance in different maize hybrids during the stress and recovery phases, and (c) to explore the tolerance mechanism of maize hybrids to combined salinity and waterlogging stresses.

## 2. Materials and Methods

### 2.1. Experimental Design and Plant Cultivation

A pot experiment was conducted in a greenhouse at the Department of Agronomy, University of Agriculture (Faisalabad Pakistan) during summer 2017. Four maize hybrids, i.e., FH-1231, FH-949, MALKA-2016, and DK-6142 were exposed to four different treatments, i.e., the control (no stress), NaCl (salinity with 10 dSm^−1^), WL (waterlogged conditions with 3 cm flooding), and NaCl + WL (combined). The maize hybrids were selected in a preliminary experiment based on germination and seedling growth data. All of these hybrids are commonly grown by local farmers in Punjab-Pakistan. FH-1231, FH-949, and MALKA-2016 were procured from the Ayub Agricultural Research Institute (AARI), Faisalabad Pakistan, and DK-6142 was procured from Monsanto, Pvt. Pakistan. The hybrid FH-949 was developed by crossing two inbred lines F-165 (male) and F-271 (female) at Maize Research Station, AARI, Faisalabad Pakistan. Each pot was filled with 7 kg of soil, and 10 seeds were sown in each pot. Well-sieved sandy clay loam soil was used to fill the pots, and 5, 1.4, and 0.9 g of NPK were applied per pot. After seed emergence, the plants were thinned to six plants per pot. After 15 days of seed emergence, the salt and waterlogging treatments were applied. The first sampling was performed after 7 days of treatment implementation (22 DAS) for morpho-physiological attributes. After sampling, normal conditions were then provided for recovery. 

### 2.2. Measurement and Physiological Analysis

Two samplings were performed after 22 and 30 DAS. The seedlings were heavily irrigated to soften the soil and were carefully harvested to avoid any root damage and were then washed. Root length was assessed with a meter rod. The shoot length of uprooted seedlings was clipped for shoot cutting, and shoot length was measured by using a meter rod and averaging. The uprooted seedlings were washed and dried in the shade for 20 min. Then, an electronic weighing balance was used to measure the fresh weights of the roots and shoots. A scale meter was used to measure the maximum leaf width/seedlings, and an average was taken. For the measurement of root and shoot dry weights, the plant roots were separated with a knife, sundried for three days, and oven-dried at 68 °C until constant weight [27]. After that, the dry weight was measured to calculate the root dry weight of each plant. 

The chlorophyll and carotenoid contents were assessed with a spectrophotometer, as described previously by Peizhou et al. [30]. Fresh leaves of plants were ground in 5 mL of 80% acetone for the determination of chlorophyll contents. The filtered solution was centrifuged at 13,000 rpm for 20 min at 4 °C. The chlorophyll a (Chl a), chlorophyll b (Chl b), and carotenoid concentrations were determined at 665, 649, and 470 nm, respectively, with the help of a spectrophotometer (UV-4000, OR1, Germany) [30]. The Chl a, Chl b, total chlorophyll, and carotenoids contents were calculated using the following equations:

Chlorophyll ‘a’ = 11.75 A_665_ − 2.350 A_649_

Chlorophyll ‘b’ = 18.61 A_649_ − 3.960 A_665_

Total Chlorophyll = Chlorophyll a + Chlorophyll b

Total Carotenoid = (1000 A_470_ − 2.270 Chl a − 81.4 Chl b)/227

### 2.3. Biochemical Parameters

#### 2.3.1. Determination of Reactive Oxygen Species (ROS)

The H_2_O_2_ contents from maize leaves were calculated as suggested by Velikova et al. [31] with slight modifications. Two grams of leaf tissues were extracted with 10 mL of TCA (0.1%, *w*/*v*) at 45 °C and homogenized. The homogenate mixture was centrifuged at 13,000 rpm for 12 min. To 1 mL supernatant, 2 mL of a 1 M potassium iodide solution and 1 mL of a 0.05 M sodium phosphate buffer (pH 7.0) were used. The absorption of the mixture was measured at the wavelength 390 nm using a spectrophotometer. The H_2_O_2_ contents were calculated by using an extinction coefficient (o) of 0.28 mM^−1^ cm^−1^ and expressed as mg/g FW.

#### 2.3.2. Antioxidant Enzyme Activities

To prepare the standards for various antioxidant enzyme extraction, fresh leaves (0.4 g) were ground by using a pestle and mortar in 10 mL of a 50 mM phosphate buffer (pH 7.8). The homogenized mixture was centrifuged at 14,000 rpm for 25 min at 4 °C, and the supernatant was used for determination of the enzyme.

Catalase activity (CAT) in fresh leaves was measured by the method described by Aebi [32] with some modifications. In a 10 mL tube, 0.4 mL of an enzyme solution and 0.4 mL of a phosphate buffer were added and pre-heated at 28 °C in a water bath for 4 min. Then, 0.6 mL of a (100 mM H_2_O_2_) solution was added to a 10 mL tube. To neutralize the enzyme solution, the control tube was heated in a boiling water bath for 5 min. Absorbance at the wavelength of 240 nm was calculated at intervals of 1 min for 3 min. One unit of enzyme activity (U) was a decrease of 0.1 for A240 within 1 min. Peroxidase (POD) activity was assayed by using the guaiacol method as described by Pütter [33] with slight changes. The mixture prepared from this technique was the 50 mM phosphate buffer (pH 7.0), which contains 10 mM guaiacol and 5 mM H_2_O_2_. This reaction mixture was first pre-heated at 20 °C in a water bath. After centrifugation, in a 10 mL tube, 2.8 mL of the reaction solution and 0.2 mL of the enzyme were mixed. Absorbance was recorded at the wavelength of 470 nm at an interval of 1 min once and determined for 4 min continuously. The superoxide dismutase (SOD) activity was measured using the method described by Dhindsa et al. [34]: fresh leaves (0.2 g) were ground in 2 mL of the 50 mM phosphate buffer (pH 7.8) with the precooled pestle and mortar. The homogenized mixture was centrifuged (13,000 rpm) at 5 °C and the supernatant was stored at 5 °C. The SOD activity was assayed by its ability to reduce the photochemical reduction of nitrob1ue tetrazolium. The test tubes with the assayed mixture (3 mL of the reaction buffer, 0.4 mL of methionine, 0.2 mL of the enzyme extract, and equal amounts of Na_2_CO_3_) were heated in light with 15 W inflorescent lamps for 20 min. Absorbance was measured with the blank and read at the wavelength of 560 nm using a spectrophotometer. The activity of ascorbate peroxidase (APX) was carried out as reported by Nakano and Asada [35] with some modifications. The reaction mixture was prepared by mixing 50 mM phosphate buffer (pH 7.0), 0.25 mM ascorbic acid, 1 mM H_2_O_2_, and 0.1 mM EDTA. In a 10 mL centrifuged tube, 2.8 mL reaction solution and 0.2 mL enzyme solution were mixed immediately, and the absorbance was measured at 290 nm at one-minute intervals, and the measurement continued for 4 min.

### 2.4. Gene Expression Analysis

The expression levels of the antioxidant enzyme genes including catalase isomer 3 (CAT) and peroxidase 39 isoform X1 (POD) were assessed in the four maize hybrids using quantitative real-time PCR (qRT-PCR) analysis. Total RNA and cDNA synthesis were performed from plant tissues following the protocols of the RNeasy Plant Mini kit and Reverse Transcription kit (Qiagen, Hilden, Germany), respectively. According to the procedures of QuantiTect SYBR Green PCR kit (Qiagen, Hilden, Germany), the PCR reactions were prepared in triplicates. The amplification circumstances were set up as follows: 95 °C for 10 min, and 40 cycles of 95 °C for 20 s, 60 °C for 30 s, 72 °C for 2 min, and 72 °C for 4 min. A melt-curve analysis was performed in order to verify the amplification specificity. Specific primers for the *CAT* and *POD* genes [36] were utilized for the amplification process. The *Actin2* gene was utilized as a housekeeping gene [36], and the relative gene expression levels were assayed following the 2^−ΔΔCt^ method.

### 2.5. Experimental Design and Statistical Analysis

The experiment was laid out in a completely randomized design with four replications. The data collected were analyzed statistically by using two-factor factorial experiments and subjected to Fisher’s analysis of variance technique. The treatments’ means were compared at the 5% probability level according to Tukey’s HSD (honestly significant difference) test.

## 3. Results

### 3.1. Plant Growth and Development

#### 3.1.1. Root and Shoot Length

**Figure 1:**
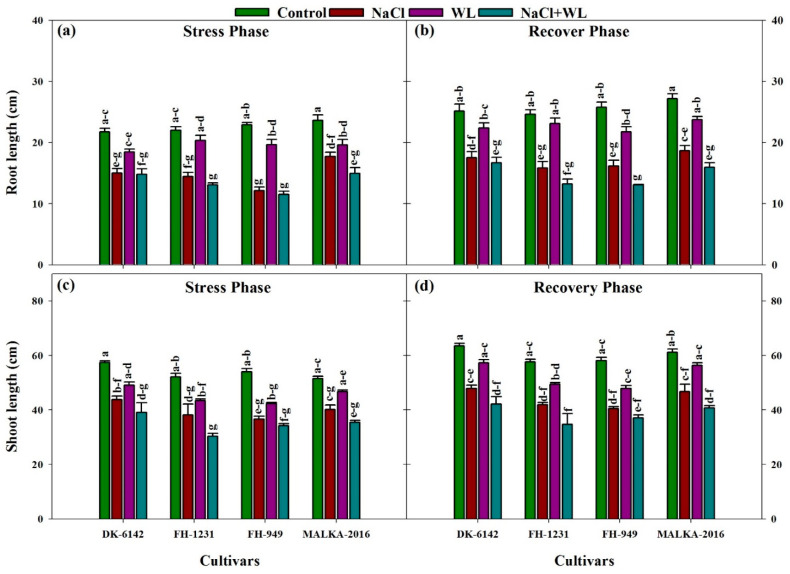
Individual and interactive effects of salinity and waterlogging on the shoot and root lengths of four maize hybrid cultivars during the stress (22 DAS) and recovery (30 DAS) phases. Left and right sides of the figure are representing stress phase and recovery phase respectively. Error bars denote the standard error of three replications. Bars with the same letters do not differ significantly at *p* ≤ 0.05.

Salinity, waterlogging, and the cultivars exhibited a significant effect on the root and shoot lengths of maize (Figure 1). Compared with the control, the root length of maize was substantially reduced in response to the stress conditions. The combined application of salinity and waterlogging was more destructive compared with their individual effects, and the negative effects of salinity alone were more obvious than that of waterlogging alone. Combined salinity and waterlogging reduced the root length by 31–49% compared with the control. Only salinity or waterlogging reduced the root growth by 30–47% and 9–24%, respectively, compared to the control (Figure 1a). Among the cultivars, larger reductions in root growth were reported for FH-1231 and FH-949, which were 20–48% higher than that of DK-6142 and MALKA-2016. The higher root length under the control and stress conditions was recorded in DK-6142, followed by MALKA-2016. In the recovery phase, root length differed significantly with stress treatments and cultivars (Figure 1b). The root length of DK-6142 was higher than that of the other cultivars. Irrespective of cultivars, the root length of maize cultivars at 30 DAS was increased by 87%, 85%, 86%, and 82% under the control, salinity, waterlogging, and combined salinity and waterlogging treatments, respectively, compared with those at 22 DAS. Moreover, under waterlogging stress, the root length was restored to the maximum level, followed by sole salinity, and combined salinity and waterlogging. 

Correspondingly, salinity stress caused a greater reduction in shoot length than waterlogging. Shoot length under salinity, waterlogging, and combined stresses were reduced (as % of controls) by 24%, 14%, and 30% in DK-6142; 27%, 19%, and 42% in FH-1231; 32%, 22%, and 36% in FH-949; and 22%, 12%, and 31% in MALKA-2016, respectively, compared with the control (Figure 1c). Moreover, in the recovery phase, the DK-6142 and MALKA-2016 cultivars performed better than other cultivars. Regardless of the cultivars, the shoot length of maize under waterlogging alone at 30 DAS was 84–90% higher than those under salinity alone or combined salinity and waterlogging, indicating the faster recovery of maize after waterlogging stress (Figure 1d).

#### 3.1.2. Root and Shoot Fresh Weights

**Figure 2:**
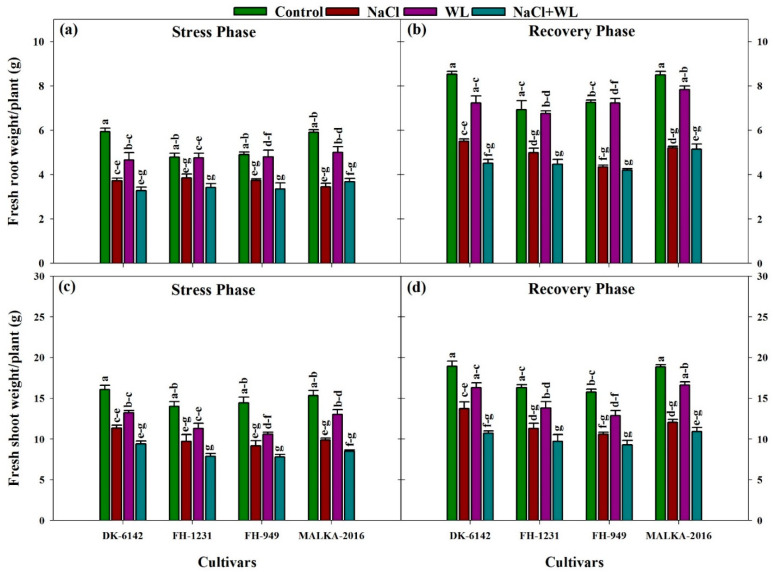
Individual and interactive effects of salinity and waterlogging on root and shoot fresh weight (g) of four maize cultivars during the stress (22 DAS) and recovery (30 DAS) phases. Left and right sides of the figure are representing stress phase and recovery phase respectively. Error bars denote the standard error of three replications. Bars with same letter do not differ significantly at *p* ≤ 0.05.

The fresh weight of roots and shoots of maize cultivars differed significantly under salinity and waterlogging stresses (Figure 2). Compared with the control, salinity, waterlogging, and combined salinity and waterlogging reduced the root fresh weight of all cultivars by 23–49%, 1–21%, and 28–44%, respectively. In general, salinity caused a greater reduction in root fresh weight compared to waterlogging stress. Among the cultivars, the stress-induced inhibitory effect was more prominent in FH-1231 than that in other cultivars (Figure 2a). In the recovery phase, DK-6142 performed better than all other cultivars. Regardless of the cultivars, the root fresh weight at 30 DAS under waterlogging stress was significantly higher (72%) compared with salinity alone or combined salinity and waterlogging (Figure 2b). Furthermore, the fresh weights of the shoot were also significantly reduced under stress conditions in all cultivars. Averaged across cultivars, a maximum reduction was recorded under combined salinity and waterlogging stress compared with salinity and waterlogging alone. Among the cultivars, the shoot fresh weight of DK-6142 was higher under stress conditions than other cultivars (Figure 2c). In the recovery phase, the negative impact of stress treatments on shoot weight was substantially reduced. Regardless of the cultivars, the root fresh weight increased at 30 DAS under the control and stress conditions compared with that recorded at 22 DAS. Compared with other stress treatments, the maximum increase (as % of the stress phase, 22 DAS) under waterlogged stress was 83% (Figure 2d).

#### 3.1.3. Dry Weight of Roots and Shoots

**Figure 3:**
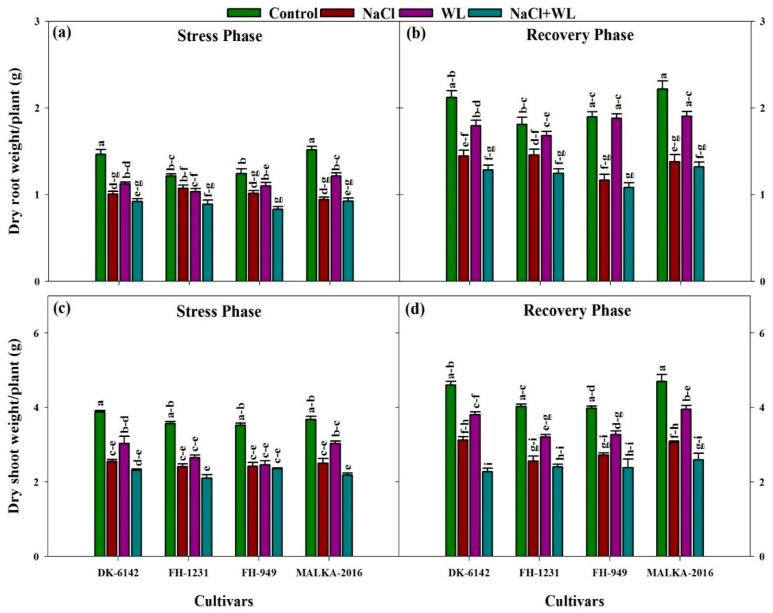
Individual and interactive effects of salinity and waterlogging on the dry weights of roots and shoots of four maize cultivars during the stress (22 DAS) and recovery (30 DAS) phases. Left and right sides of the figure are representing stress phase and recovery phase respectively. Error bars denote the standard error of three replications. Bars with same letter do not differ significantly at *p* ≤ 0.05.

There was a significant difference between all treatments for the root and shoot dry weights. Compared with the control, the root dry weight decreased under the stress treatments. Averaged across cultivars, the maximum reduction (29%) was recorded under combined salinity and waterlogging stress. Moreover, the only salinity treatment reduced the root dry weight more severely compared to the only waterlogging treatment. Under stress conditions, the relative reduction in root dry weight was significantly higher for the FH-1231 and FH-949 cultivars compared to the DK-6142 and MALKA-2016 cultivars (Figure 3a). In the recovery phase, the root dry weight increased (% of stress phase) by 74%, 65%, 72%, and 62% under the control, salinity, waterlogging, and combined salinity and waterlogging stress, respectively, regardless of the cultivar (Figure 3b). Among the stress treatments, upon averaging the cultivars, a larger reduction in shoot dry weight (38% of control) was recorded under combined salinity and waterlogging stress compared to only salinity and waterlogging stress. Among the cultivars, a minimum reduction in shoot dry weight was recorded for MALKA-2016 (Figure 3c). In the recovery phase, the dry weight of shoot under stress treatments was increased, and the maximum increase (72% compared to 22 DAS) was found under only waterlogging stress (Figure 3d).

#### 3.1.4. Leaf Width and Number of Leaves per Plant

**Figure 4:**
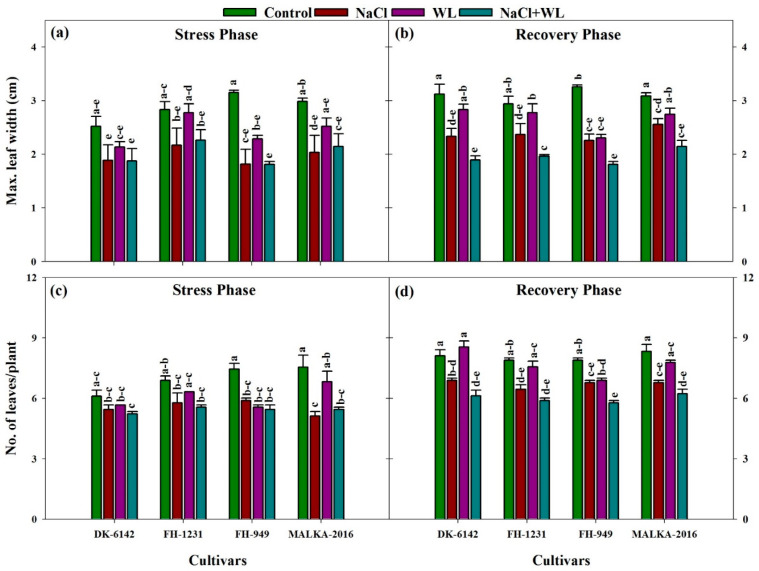
Individual and interactive effects of salinity and waterlogging on maximum leaf width and number of leaves/plant of four maize cultivars during the stress (22 DAS) and recovery (30 DAS) phases. Left and right sides of the figure are representing stress phase and recovery phase respectively. Error bars denote the standard error of three replications. Bars with same letter do not differ significantly at *p* ≤ 0.05.

Both salinity and waterlogging stresses reduced the leaf width and the number of leaves of maize; however, such reductions under salinity alone were more severe compared with waterlogging alone (Figure 4a). Among the cultivars, leaf width was slightly reduced in DK-6142 and MALKA-2016; however, the reduction was more pronounced in the FH-949 cultivar. Moreover, in the recovery phase, DK-6142 and MALKA-2016 performed well compared with the other cultivars (Figure 4b).

Salinity and waterlogging as individual and concurrent stresses significantly hindered the number of leaves per plant. However, a significantly smaller number of leaves were recorded under the combined stresses than for salinity or waterlogging alone (Figure 4c). Among the cultivars, MALKA-2016 and DK-6142 recorded a greater number of leaves compared to the FH-1231 and FH-949 cultivars. The number of leaves following the recovery phase from the stress conditions was increased significantly to that of the stress phase. Additionally, the maximum increase was recorded under the waterlogged condition than that under other stress treatments (Figure 4d).

### 3.2. Physiological Parameters

#### 3.2.1. Chlorophyll and Carotenoids Contents

**Figure 5:**
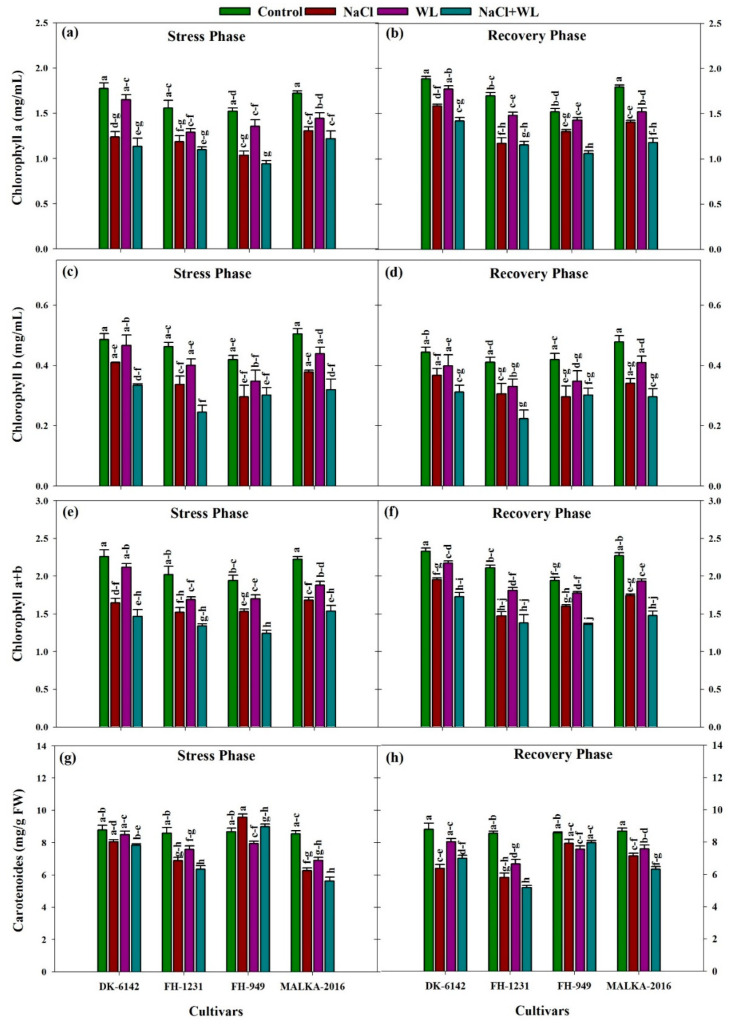
Individual and interactive effects of salinity and waterlogging stress on chlorophyll ‘a’, ‘b’, ‘a + b’ (mg/mL), and carotenoid content of four maize cultivars during the stress (22 DAS) and recovery (30 DAS) phases. Left and right sides of the figure are representing stress phase and recovery phase respectively. Error bars denote the standard error of three replications. Bars with same letter do not differ significantly at *p* ≤ 0.05.

Significant differences in the chlorophyll contents of maize cultivars were recorded under the influence of salinity and waterlogging stresses. The lowest Chl a content was observed in the combined salinity and waterlogging stress, which was about 29–38% lower than that in the control (Figure 5). During the recovery phase, the Chl a content under stress treatments improved significantly and the maximum recovery in Chl a content was observed in waterlogging stress alone, which was about 7–95% higher than the stress phase (22 DAS), depending on the cultivars (Figure 5). Similarly, salinity and waterlogging stress significantly decreased the Chl b content. The highest decrease in Chl b content was found in combined salinity and waterlogging stress, which was about 25-41% compared to the control. Moreover, during the recovery phase, an increment in Chl b content was recorded under all stress treatments.

Salinity and waterlogging stress significantly reduced the Chl a + Chl b contents of maize leaves (Figure 5). Under the combined salinity and waterlogging stress, the Chl a + Chl b contents decreased by 32–47% compared with the control, depending on the cultivars. Both the Chl a + Chl b contents following the recovery phase increased significantly under stress conditions. The maximum increase (% of stress phase) of 92% was recorded under sole waterlogging stress. For the cultivars, the chlorophyll contents in DK-6142 and MALKA-2016 were higher in all treatments compared with that in the FH-1231 and FH-949 cultivars. In general, the inhibitory effects were more prominent in combined salinity and waterlogging conditions than their individual effects.

The carotenoids content was also reduced under stress conditions, but the negative effects of combined salinity and waterlogging stress were more severe than for stresses applied alone for some cultivars. For individual stress, the negative effects of salinity were more pronounced compared that of the waterlogged condition in some cultivars. In the recovery phase, significant variations were observed among the stress treatments, with the maximum increase (about 84%) in waterlogging stress. 

#### 3.2.2. Hydrogen Peroxide Contents

**Figure 6:**
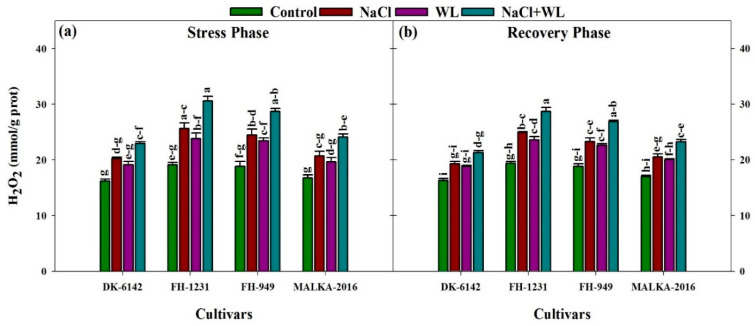
Individual and interactive effects of salinity and waterlogging on the H_2_O_2_ concentrations of four maize cultivars during the stress (22 DAS) and recovery (30 DAS) phases. Left and right sides of the figure are representing stress phase and recovery phase respectively. Error bars denote the standard error of three replications. Bars with same letter do not differ significantly at *p* ≤ 0.05.

When plants were exposed to individual and combined salinity and waterlogging stresses, the H_2_O_2_ content in all cultivars increased significantly, with the most significant increase of 24–43% under combined salinity and waterlogging stress, depending on the cultivars (Figure 6). The FH-1231 cultivar had higher H_2_O_2_ content compared with the other cultivars. In the recovery phase, the H_2_O_2_ content decreased in all treatments (Figure 6). Additionally, the DK-6142 cultivar performed well during the stress and recovery phases compared with all other cultivars.

### 3.3. Antioxidant Enzymes

**Figure 7:**
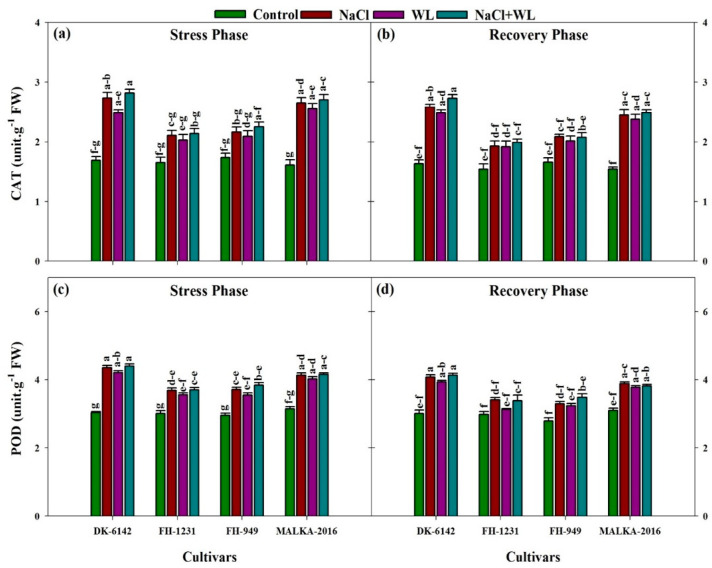
Individual and interactive effects of salinity and waterlogging on catalase (CAT) and peroxidase (POD) activity in four maize cultivars during the stress (22 DAS) and recovery (30 DAS) phases. Left and right sides of the figure are representing stress phase and recovery phase respectively. Error bars denote the standard error of three replications. Bars with same letter do not differ significantly at *p* ≤ 0.05.

**Figure 8:**
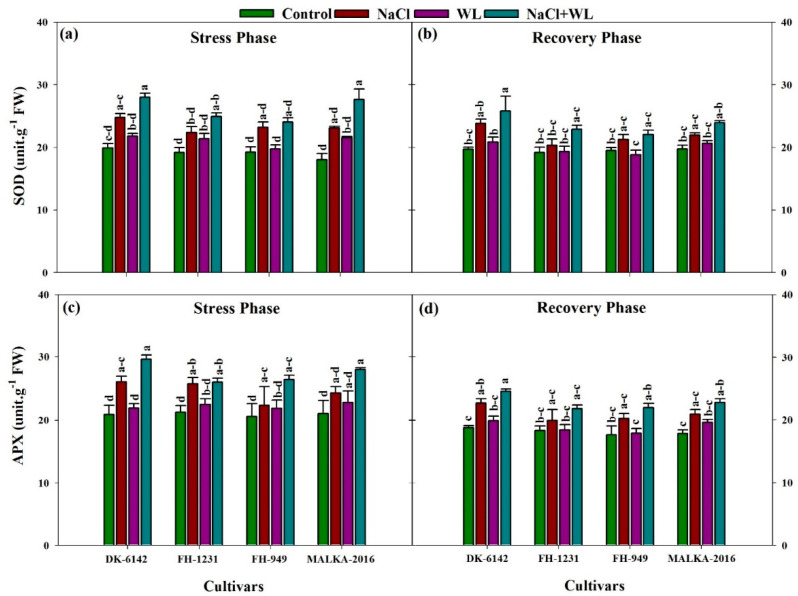
Individual and interactive effects of salinity and waterlogging on superoxide dismutase and ascorbate peroxidase activity in four maize cultivars during the stress (22 DAS) and recovery (30 DAS). Left and right sides of the figure are representing stress phase and recovery phase respectively. phases. Error bars denote the standard error of three replications. Bars with same letter do not differ significantly at *p* ≤ 0.05.

The activities of the antioxidant enzymes (CAT, POD, SOD, and APX) in maize leaves increased significantly under individual and combined application of salinity and waterlogging stress for all cultivars (Figure 7 and Figure 8). The CAT activities were increased by 24–65%, 20–58%, and 29–67% under salinity, waterlogging, and combined salinity and waterlogging, respectively, compared with their respective controls. In the recovery phase, a substantial decline in CAT activity was reported for all stress treatments and cultivars. In the stress and recovery phases, more CAT content was recorded in DK-6142 and MALKA-2016 compared to FH-1231 and FH-949 (Figure 7). The POD activity was also affected significantly by stress treatments and cultivars. A substantial increase in POD activity was reported for all stress treatments, with the maximum increase (% of control) in the range of 23–45% under combined salinity and waterlogging stress. In the recovery phase, the POD activity was decreased significantly compared with the stress phase. 

A significant variation in SOD activity was observed under individual and concurrent effects of stress treatments. Averaged across cultivars, a greater increase in SOD activity in the range of 23–41% was observed under the concurrent effect of these stresses compared with the control plants (Figure 8). In the recovery phase, a substantial decline in SOD activity was observed for the stress treatments. Among the cultivars, better performance in the DK-6142 cultivar was recorded compared to the other cultivars. The APX activity of all four maize cultivars increased under stress conditions, and their activity was higher under combined salinity and waterlogging stress compared with salinity and waterlogging alone. The APX activity in the DK-6142 and MALKA-2016 cultivars was higher compared to that in the FH-1231 and FH-949 cultivars. After the recovery phase, the plants showed a decreasing trend in APX activity. 

### 3.4. Gene Expression Analysis

**Figure 9:**
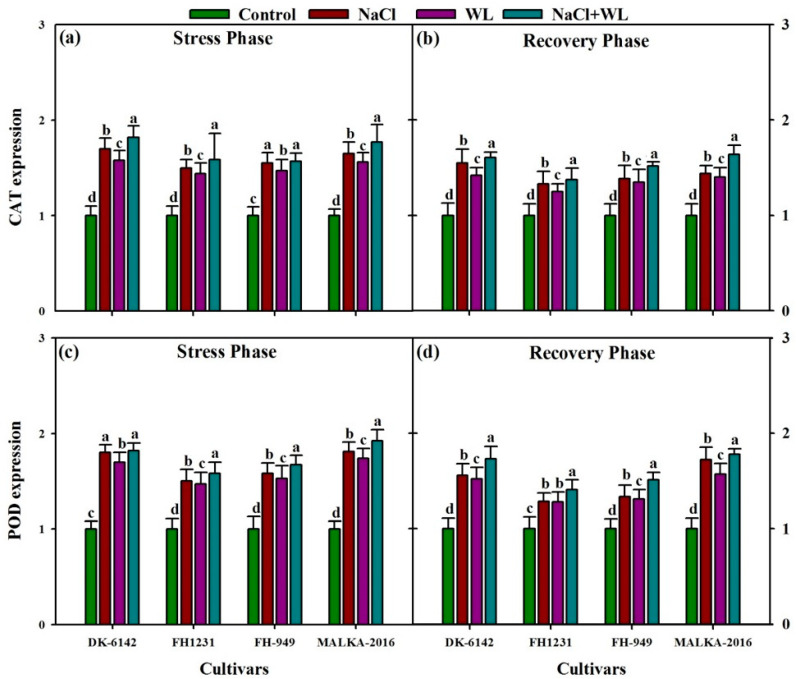
Individual and interactive effects of salinity and waterlogging on the expression of antioxidant enzyme genes including catalase isomer 3 (CAT) and peroxidase 39 isoform X1 (POD) in four maize cultivars during the stress (22 DAS) and recovery (30 DAS) phases. Left and right sides of the figure are representing stress phase and recovery phase respectively. Error bars denote the standard error of three replications. Bars with same letter do not differ significantly at *p* ≤ 0.05.

The expression levels of the two antioxidant genes (*CAT* and *POD*) in maize leaves increased significantly under individual and combined application of salinity and waterlogging stress for all cultivars (Figure 9). The expression levels of *CAT* gene were enhanced by 50–70%, 44–58%, and 57–82% under salinity, waterlogging, and combined stress, respectively, compared with their respective controls. In the recovery phase, a substantial decline in *CAT* expression levels was reported for all stress treatments and cultivars. In the stress and recovery phases, a higher expression level of the *CAT* gene was recorded in DK-6142 and MALKA-2016 compared to that in FH-1231 and FH-949 (Figure 9). The expression level of the *POD* gene was also affected significantly by the stress treatments and cultivars. A substantial increase in the *POD* gene expression level was recorded for all stress treatments, with a maximum increase of 58–92% under combined salinity and waterlogging stress. In the recovery phase (30 DAS), the expression level of the *POD* gene decreased significantly compared with the stress phase (22 DAS). 

## 4. Discussion

This study, which was conducted under controlled conditions, examined the individual and simultaneous effects of salinity and waterlogging stress on the morpho-physiological, biochemical, and molecular responses of maize crop. The exposure of maize cultivars to salinity and waterlogging stress led to a significant reduction in growth traits, i.e., root and shoot lengths, dry and fresh weights, leaf widths, and number of leaves per plant. However, the combined salinity and waterlogging stresses caused greater reductions than salinity and waterlogging stresses alone. These results are consistent with previously published findings [37,38]. A greater accumulation of Na^+^ ions leads to a decrease in root and shoot lengths [39]. Compared with individual salinity and waterlogging stresses, a greater increase in Na^+^ content under combined salinity and waterlogging was reported elsewhere [40]. The increase in Na^+^ content under the combined salinity and waterlogging significantly affects plant metabolism, reduces the efficiency of photosynthesis, causes more necrotic and chlorotic leaves, and ultimately leads to a serious decline in growth traits [40]. The combined effect of salinity and waterlogging stress also resulted in increases in Mn and Fe contents in the soil [40]; excessive Mn adversely affected the photosynthetic apparatus, reduced nutrient absorption, and thus reduced the plant growth [41]. More necrotic and chlorotic leaves under excessive Mn conditions were also reported by Führs et al. [42]. The combined presence of these elements (Mn + Fe) can cause excessive accumulation of ROS [43] and can reduce chlorophyll content and photosynthesis efficiency [40], which eventually led to a serious decline in plant growth. Under stress conditions, high concentrations of ROS can lead to turgidity loss and membrane damage [10,44]. In this study, combined salinity and waterlogging also resulted in higher ROS production and reduced chlorophyll content compared to the sole application of salinity or waterlogging (Figure 6). During the recovery phase, a greater recovery of growth characteristics after waterlogging may also be attributed to the increase in chlorophyll content and the decrease in ROS production. Furthermore, the better growth of DK-6142 in the stress and recovery phases may be attributed to the reduced ROS production and higher chlorophyll content. In addition to generating ROS, combined salinity and waterlogging can also reduce the oxygen availability to a great extent [12,22,45]. This situation can lead to a greater reduction in biomass accumulation. Under sole salinity, the decrease in growth traits, and root and shoot lengths may be due to inappropriate Na^+^/K^+^ ratios, as higher values of Na^+^/K^+^ in plant tissue impairs the transport of K^+^ and Ca^2+^, disturbs plant metabolism, and leads to reduced plant growth [46]. Under salinity stress, reduced leaf initiation, poor cell expansion, lower intermodal distance, and higher risk of leaf abscission also result in reduced shoot growth [11,47,48]. The increase in Na^+^ ions, the decrease in chlorophyll content, and the overproduction of ROS are the main reasons for the decrease in maximum leaf width [39]. Under individual waterlogging stress, the decrease in root and shoot lengths may be due to the change in soil redox potential, O^-^_2_ depletion, and ROS production [24,49,50]. The greater reduction in leaf widths and fewer leaves per plant were mainly attributed to decreased water potential and stomatal conductance, increased root senescence, reduced shoot and root growth [51], and decreased gaseous exchange between air and soil [52,53].

Chlorophyll is a main part of photosynthesis and is imperative for plant physiological processes [54]. This study showed that salinity and waterlogging, as individual and combined stresses, resulted in significant decreases in chlorophyll content; however, such a reduction was more severe under the combined salinity and waterlogging stress (Figure 5). These findings are consistent with the results of previous studies [40,55], where the authors reported greater decreases in chlorophyll content under the combined salinity and waterlogging conditions. The reductions under combined salinity and waterlogging stresses were mainly due to the reduced photosynthesis rate under these stresses [55]. At high salinity, waterlogging stress hinders the efficiency of photosystem II and reduces the photosynthesis efficiency of plants [55]. Under waterlogging stress, oxygen deficiency in the roots reduces aerobic respiration and markedly limits ATP production [56]. This reduction seriously hampers plant metabolism and ultimately leads to a reduction in chlorophyll content and photosynthetic efficiency. Reductions in ATP production (2–3 fold) under combined salinity and waterlogged conditions were reported by Zeng et al. [40]. As mentioned above, under the combined salinity and waterlogging stress, excessive Mn and Fe disturb Na^+^/K^+^ homeostasis and ultimately lead to a decrease in chlorophyll content. A large increase in Na^+^ and Cl^-^ concentration was also reported under combined salinity and waterlogging stress [51,57]; a greater accumulation of Na^+^ and Cl^-^ ions caused a marked reduction in chlorophyll content [58]. Under salinity stress, an inadequate supply of K is the main reason for the growth reduction in maize, which reduces the photosynthetic rate and increases oxidative damage [13]. Under salt stress, Na^+^ is a principally toxic ion, which interferes with K uptake, leads to disturbance in stomatal conductance, and causes water loss and necrosis [12]. The unbalanced ratios of Na^+^ and K^+^ under saline conditions severely reduce the K^+^ content in the leaves and roots of maize [46]. Moreover, in the initial stages of salt stress, high concentrations of Na^+^ can also disturb the calcium (Ca^2+^) levels, and the transport of Ca^2+^ to young leaves is impaired [59]. A certain amount of Ca^2+^ is required to maintain the integrity of the cell membrane, and therefore, leaf expansion was reduced with lower Ca^2+^ content in the shoot tissues of maize under salinity stress [59]. Under waterlogged conditions, a lack of oxygen can cause a significant decrease in the net photosynthetic rate [60]. The decrease in photosynthetic rate is attributed to the closure of stomata, to decreased chlorophyll content, and to leaf senescence [61]. In the present study, the better performances of DK-6142 and MALKA-2016 were due to the lower reduction in chlorophyll content during the stress period and to the greater increase in chlorophyll content during the recovery period (Figure 5).

The stress treatment has a significant effect on the carotenoid content, with the maximum reduction under combined salinity and waterlogging stress. Reductions in carotenoid content under stress conditions have been discussed in earlier studies [12,62]. Carotenoids play an essential role in the quenching of singlet oxygen, and their relative levels in the cultivar(s) may indicate their tolerance [63]. 

Salinity and waterlogging treatments significantly affect H_2_O_2_ content, and the highest H_2_O_2_ contents were recorded under combined salinity and waterlogging stress, compared with salinity and waterlogging alone. Similar findings were reported by Duhan et al. [64], who reported that combined salinity and waterlogging increased the H_2_O_2_ content in the range of 43–75% in pigeon pea. Compared with salinity and waterlogging alone, the H_2_O_2_ contents were also higher in a wheat plant affected by combined stresses [37]. A greater accumulation of ROS is the cause of membrane damage, which is due to loss of turgidity and reduced growth. ROS are very reactive and can interrupt normal cellular activities through oxidative damage [26]. Previous studies have reported increased ROS levels under salinity stress [14,65]. According to Mittler [66], an increase in salinity level significantly increases the H_2_O_2_ content. Similarly, under waterlogged conditions, a greater accumulation of H_2_O_2_ was also recorded in previous studies [23,24]. During the recovery phase, the H_2_O_2_ content decreased significantly in all stress treatments. Among the cultivars, the maximum recovery in the DK-6142 cultivar was due to the decreased level of H_2_O_2_ (Figure 6).

Salinity and waterlogging, as individual or concurrent stresses, can lead to oxidative damage in plants. Reducing the accumulation of ROS and detoxifying oxidative stress are the main mechanisms for coping with abiotic stress. The accumulation of antioxidant enzymes plays an important role in adapting plants to the adverse effects of ROS [67]. Salinity and waterlogging stress can cause higher ROS, which may activate the antioxidant defense system. In the present study, the activities of CAT, POD, SOD, and APX in all maize cultivars were enhanced under stress conditions. The expression levels of the antioxidant genes (*CAT* and *POD*) were also upregulated under stress conditions. The higher activities of the CAT enzyme were found under salinity, and combined salinity and waterlogging stress compared with the control or waterlogging alone. These findings are consistent with the results of Duhan et al. [64], which reported a 41–83% increase in CAT enzyme activity in pigeon pea under combined salinity and waterlogging stress. Similarly, Haddadi et al. [68] reported that under, combined salinity and waterlogging stress, the activity of CAT was increased by 93–112% when compared with the control. The higher activity of CAT helps to minimize the adverse effects of salinity and waterlogging [27]. The CAT enzyme is present in peroxisomes and plays an important role in the dismutation of H_2_O_2_ into O_2_ and H_2_O [25]. In addition, POD activity was also found to increase under stress treatments, and the maximum increase was recorded under combined salinity and waterlogging stress. A similar increase in POD activity under combined salinity and waterlogging stress [64,69] and its involvement in the detoxification of ROS [27,70] has been widely reported.

In the present study, an increased SOD activity was noted for all of the cultivars under salinity and waterlogged conditions; however, a greater increase was observed under the combined occurrence of these stresses. Under combined salinity and waterlogging stress, an increase in SOD content has also been reported elsewhere [55]. A higher SOD activity can play a role in minimizing the negative effects of abiotic stresses [65,71]. APX is an important enzyme that helps in the detoxification of H_2_O_2_ [70]. In the present study, a remarkable increase in APX activity was recorded for all stress treatments and cultivars. Similar findings were also reported in previous research [68], where the combined salinity and waterlogging led to greater increases in APX activity than the individual occurrence of these stresses. Taken together, similar to the pronounced impacts of main environmental stresses on plants [72,73,74,75,76,77], salinity and waterlogging stresses revealed morpho-physio-biochemical and molecular impacts in maize.

## 5. Conclusions

The present study demonstrated that salinity and waterlogging stresses hinder the growth and physiological characteristics of all maize cultivars examined in this study. However, the effects of combined salinity and waterlogging stress were more severe compared to their individual effects. In the recovery phase, plants under only waterlogging stress showed faster and better recovery in their growth traits and chlorophyll content than salinity alone or combined salinity and waterlogging stress. Among the cultivars, the performance of DK-6142 was better, followed by MALKA-2016, during the stress as well as recovery phases. The better growth performance and stress tolerance of DK-6142 was mainly attributed to the chlorophyll content and the more effective antioxidant defense system, which ameliorates the negative impact of salinity and waterlogging stresses.

## Data Availability

All of the data supporting the findings of this study are included in this article.
